# Type‐Independent 3D Writing and Nano‐Patterning of Confined Biopolymers

**DOI:** 10.1002/advs.202207403

**Published:** 2023-02-24

**Authors:** Un Yang, Byunghwa Kang, Moon‐Jung Yong, Dong‐Hwan Yang, Si‐Young Choi, Jung Ho Je, Seung Soo Oh

**Affiliations:** ^1^ Department of Materials Science and Engineering Pohang University of Science and Technology (POSTECH) 77 Cheongam‐Ro, Nam‐Gu Pohang Gyeongbuk 37673 South Korea; ^2^ Nanoblesse 85‐11 (4th fl.) Namwon‐Ro Pohang Gyeongbuk 37883 South Korea; ^3^ Institute for Convergence Research and Education in Advanced Technology (I‐CREATE) Yonsei University 85 Songdogwahak‐ro Yeonsu‐gu Incheon 21983 South Korea

**Keywords:** 3D writing, biopolymers, nanoscale confinement, solvent‐exclusive evaporation, sub‐micron resolution

## Abstract

Biopolymers are essential building blocks that constitute cells and tissues with well‐defined molecular structures and diverse biological functions. Their three‐dimensional (3D) complex architectures are used to analyze, control, and mimic various cells and their ensembles. However, the free‐form and high‐resolution structuring of various biopolymers remain challenging because their structural and rheological control depend critically on their polymeric types at the submicron scale. Here, direct 3D writing of intact biopolymers is demonstrated using a systemic combination of nanoscale confinement, evaporation, and solidification of a biopolymer‐containing solution. A femtoliter solution is confined in an ultra‐shallow liquid interface between a fine‐tuned nanopipette and a chosen substrate surface to achieve directional growth of biopolymer nanowires via solvent‐exclusive evaporation and concurrent solution supply. The evaporation‐dependent printing is biopolymer type‐independent, therefore, the 3D motor‐operated precise nanopipette positioning allows in situ printing of nucleic acids, polysaccharides, and proteins with submicron resolution. By controlling concentrations and molecular weights, several different biopolymers are reproducibly patterned with desired size and geometry, and their 3D architectures are biologically active in various solvents with no structural deformation. Notably, protein‐based nanowire patterns exhibit pin‐point localization of spatiotemporal biofunctions, including target recognition and catalytic peroxidation, indicating their application potential in organ‐on‐chips and micro‐tissue engineering.

## Graphical abstract

Biopolymer‐based three‐dimensional (3D) architectures hold great potential to control and mimic living cells. This work demonstrates direct 3D writing technique to generate biopolymeric nano‐architectures with precisely‐controlled size and desired geometry. The 3D writing and nano‐patterning is biopolymer type‐independent with no functional loss, allowing pin‐point localization of spatiotemporal biofunctions toward applications in biotechnology, such as organ‐on‐chips and micro‐tissue engineering.

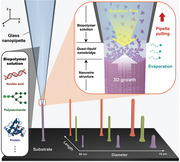

## 1 Introduction

At the submicron scale, the availability of biopolymer‐based diverse three‐dimensional (3D) architectures enables the precise control of cells and tissues for in vitro and in vivo applications.^[^ ^1^ ^]^ Biological polymers, such as nucleic acids, proteins, and glycans, which are essential building blocks of biological systems, can be well organized to build complex nanostructures.^[^ ^2^, ^3^ ^]^ The replication of their structural properties and molecular functions at the cell and tissue levels is applicable in a wide range of biomedical fields, including regenerative medicine,^[^ ^4^ ^]^ organ manufacturing,^[^ ^5^ ^]^ and microfluidic tissue engineering.^[^ ^6^ ^]^ Furthermore, their micropatterned substrates or 3D scaffolds serve as physical or chemical cues to induce cell signaling for the modulation of cellular behaviors, such as cell adhesion, migration, and proliferation. For instance, aligned actin fibers underneath certain cells guide cellular mechanotransduction, thereby leading to directional elongation and migration of fibroblasts,^[^ ^7^ ^]^ or selective differentiation of stem cells.^[^ ^8^ ^]^ Additionally, the insertion of submicronized needles or tubes through plasma membranes facilitates the delivery of exogenous biomolecules into living cells without inducing critical cellular damage.^[^ ^9^ ^]^ The Food and Drug Administration regulates unethical clinical trials, thus, shedding a spotlight on human organ‐on‐chips that mimic cell microenvironments and simultaneously maintain tissue‐specific functions.^[^ ^10^, ^11^ ^]^ In drug development and pathological studies, organ‐mimicking models are now recognized as an innovative approach to in vitro testing,^[^ ^12^ ^]^ thereby confirming the technical need for 3D micro‐ and nano‐printing of biopolymers with controlled structures, alignments, properties, and even polymeric types.

The 3D multiscale fabrication of biopolymer architectures, however, has been eager to be type‐dependent, which is attributed to the diverse rheological and structural properties of the constituents. To date, many different biopolymers have been printed at the desired positions, mainly via nozzle‐based extrusion^[^ ^13^ ^]^ and light‐assisted stereolithography.^[^ ^14^, ^15^ ^]^ In particular, in monolithic 3D nano‐printing, their dynamic rheological behaviors become more critical for controlled shaping with nanometer precision.^[^ ^16^, ^17^ ^]^ For example, unlike fibrous proteins, globular proteins do not retain submicron shapes without physical entanglement in nozzle‐based extrusion,^[^ ^18^ ^]^ thereby limiting the printing resolution and shape fidelity.^[^ ^19^ ^]^ Certain additives, such as natural fibers (e.g., alginate and collagen) or reinforcement materials (e.g., carbon nanotubes and silver nanoparticles), can be included during extrusion,^[^ ^20^, ^21^ ^]^ however, printed nanocomposites often experience unfavorable phase separation with the formation of uneven nanostructures,^[^ ^22^, ^23^ ^]^ and functional loss of the original biopolymers. For uniform compositions, biopolymers are chemically interconnected during simultaneous 3D printing. However, bio‐inks inevitably contain crosslinking agents that are inherently toxic to biological entities, and chemical entanglement poses a substantial risk that deteriorates the functional integrity of the printed biopolymers.^[^ ^24^, ^25^ ^]^ Notably, practical applications (e.g., needle patches,^[^ ^26^ ^]^ tissue scaffolds,^[^ ^27^ ^]^ and vascular networks^[^ ^28^ ^]^) demand the construction of freestanding 3D architectures that are not influenced by substrates and their geometry. To expand the repertoire of printable biopolymers at the submicron scale, developing bioprinting techniques that are type‐independent and facilitate full control over composition and shape remains exceptionally challenging.

Therefore, in this study, we demonstrate a type‐independent 3D writing technique using a systemic combination of nanoscale confinement, evaporation, and solidification of a biopolymer solution. When a femtoliter solution is confined in an ultra‐shallow liquid interface between the tip of the fine‐tuned nanopipette and the printing point of the substrate surface, solvent‐exclusive evaporation and concurrent solution supply occurs, resulting in the directional growth of biopolymeric nanowires. As the choice of biopolymer type did not strongly influence the synchronization of solvent evaporation and solution flow, the simple control over concentrations and molecular weights of biopolymers readily assured 3D complex printing with submicron resolution. Owing to the 3D motor‐operated precise nanopipette positioning, various nanowire patterns could be fabricated with different structural complexities and biological functionalities; with a broad range of diameters (80 nm–10 µm) and lengths (≥1 µm), pillar or arch‐shaped architectures were built using numerous different biopolymers, even including catalytically‐active enzymes. Importantly, the exclusive surface modification of pre‐printed nanowires could successfully secure the structural integrity of the 3D nanowire array in diverse solvents with no functional loss of biopolymers. Particularly, pin‐point compartmentalization of protein functions can be achieved, thereby realizing the potential to analyze, support, or even stimulate spatially complex cells and tissues.

## 2 Results and Discussion

### 2.1 Fabrication and Characterization of Biopolymer Nanowires

**Figure 1:**
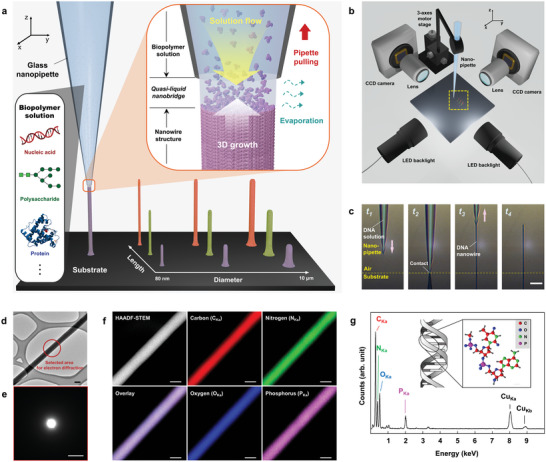
Direct 3D writing of biopolymers on a chosen substrate. a) Schematic illustration of the designed 3D printing of individual nanowires with different diameters, lengths, and biopolymers. When a quasi‐liquid nanobridge is formed between the pipette tip and the substrate surface, rapid solvent evaporation occurs at the edge. This maintains a continuous solution flow from the top (yellow arrow) and simultaneous biopolymer precipitation at the base, all of which lead to the growth of the biopolymer nanowire (white arrow). Pulling the nanopipette up (red arrow) enables continuous and directional 3D printing of desired biopolymers. b) Customized setup for biopolymer nanowire fabrication comprising 3‐axis motorized stages and optical imaging system. c) Optical microscopy images of the continuous 3D writing process. (*t*_1_) A glass nanopipette filled with DNA solution approaches a silicon (Si) substrate. (*t*_2_) The nanopipette is pulled down close to the Si substrate, forming a quasi‐liquid nanobridge in‐between. (*t*_3_) The nanopipette is subsequently pulled upward to a designed height (*h* = 40 µm), resulting in the continuous growth of the DNA nanowire. (*t*_4_) A freestanding biopolymer nanowire remains at the desired location of the substrate after separating the nanopipette. Scale bar, 10 µm. d) A bright‐field transmission electron microscopy (TEM) image of a lambda DNA nanowire. Scale bar, 100 nm. e) Selected‐area electron diffraction pattern (red circle in (d)) represents the amorphous character of the DNA nanowire. Scale bar, 1 nm^−1^. f) High angle annular dark field scanning transmission electron microscopy (HAADF‐STEM) image of a lambda DNA nanowire (top‐left) and energy dispersive X‐ray spectroscopy (EDS) overlay image of carbon (C), nitrogen (N), oxygen (O), and phosphorus (P) (bottom‐left). Each element is evenly distributed along nanowire: C (red, top‐middle), N (green, top‐right), O (blue, bottom‐middle), P (magenta, bottom‐right). Scale bars, 100 nm. g) EDS spectrum of the DNA nanowire (inset: schematic illustration showing molecular structure and composition of DNA).

Our direct 3D writing method facilitates the formation of 3D patterns consisting of individually size‐controlled nanowires formed using various biopolymers, including nucleic acids, polysaccharides, and many different proteins (**Figure** **1**a). The in situ growth of the biopolymer nanowires relies on two important procedures: nanoscale solution confinement and evaporation‐induced solidification. A femtoliter biopolymer solution is readily confined at the tip of the nanopipette, therefore, an ultra‐shallow liquid interface can be created by bridging the end of the glass nanopipette and the desired point of the substrate surface. The liquid nanobridge allows air‐driven interfacial evaporation, without the escape of solutes, while ensuring a continuous solution flow from the tip (yellow arrow). Precipitation and accumulation of biopolymers can be induced simultaneously at the base of the quasi‐liquid, thereby leading to the 3D growth of biopolymer nanowires (white arrow). When the quasi‐liquid nanobridge is preserved with no shrinkage, directional pulling‐up of the nanopipette can guide the diameter of the nanowire (*d*) to be equal to that of the nanopipette tip (*d*_t_), and the length can be eventually determined by the pulling‐up length.

Real‐time observation of our 3D writing process enabled precise positioning and controlling of nanowire growth, as implemented through a customized setup of a 3‐axes motorized stage and two imaging cameras at right (90°) angles to the same focal point (Figure 1b). As an example, a freestanding nanowire composed of pure DNAs was fabricated on a silicon surface with a predesigned diameter (*d* ≈ 500 nm) and length (*l* = 40 µm) (Figure 1c and Movie S1, Supporting Information). When filled with a DNA solution (4 mg mL^−1^ in deionized water), a nanopipette with a tip diameter of *d*_t_ = 500 nm was pulled down to the substrate (*t*_1_), and its close proximity to the surface enabled the formation of a quasi‐liquid nanobridge in between (*t*_2_). Subsequently, the nanopipette was pulled upward to a designed height (*h* = 40 µm), resulting in continuous growth of a pure DNA nanowire (*t*_3_). A rapid pulling‐off movement resulted in a freestanding DNA nanowire with a diameter and length of ≈500 nm and 40 µm, respectively, at the desired location of the substrate (*t*_4_). The growth rate of the nanowires was generally >1 µm s^−1^.

Without additives, pure biopolymers can form homogeneous nanowires through 3D writing. To investigate i) molecular arrangement and ii) spatial element distribution of biopolymers in the grown nanowires, i) transmission electron microscopy (TEM) and ii) high angle annular dark field‐scanning transmission electron microscopy (HAADF‐STEM) combined with energy dispersive X‐ray spectroscopy (EDS) characterization were performed on lambda DNA nanowires directly fabricated on a lacey carbon film supported copper (Cu) grid (Figure 1d and Figure S1, Supporting Information, and Movie S2, Supporting Information). The electron diffraction pattern in the selected area revealed the amorphous character of the DNA nanowire (Figure 1e), presumably due to the rapid packing of biopolymers at 25 °C. Nonetheless, the DNA‐composing elements in the nanowire were uniform, demonstrated by the elemental spatial distribution of C, O, N, and P via TEM‐EDS mapping (Figure 1f and Figure S2, Supporting Information). Except for Cu EDS signal originated from the grid, DNA constituents (C, O, N, and P) were mainly found in the EDS spectrum, confirming the exceptional purity of the DNA nanowires (Figure 1g).

Further simple variation of nanowire length permitted accurate quantification even at the sub‐femtomole scale by real‐time polymerase chain reaction (Figure S3, Supporting Information), after inspecting homogeneous elemental spatial distribution revealed by the STEM‐EDS of DNA nanowires. In sharp contrast to common stereolithography with harmful UV light,^[^ ^29^, ^30^ ^]^ our direct 3D writing maintains a mild processing environment, such as room temperature and ambient air, thus, resulting in no critical damage to the printed biopolymers. Irrespective of the type of printed DNA molecules, a single band was visualized in each lane of gel electrophoresis, thereby indicating full preservation of molecular integrity (Figure S4, Supporting Information).

### 2.2 Growth Behavior of Biopolymer Nanowire

**Figure 2:**
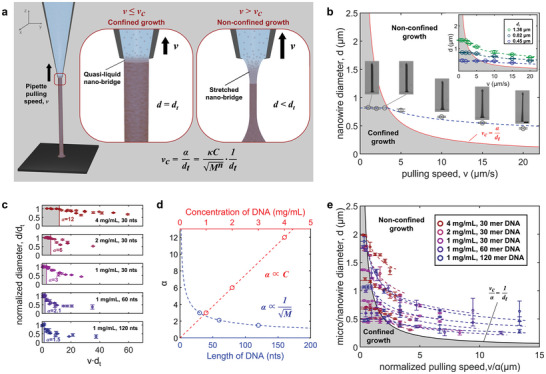
Growth dynamics of biopolymer wires. a) Schematic illustration of the biopolymer nanowire growth behavior during the 3D writing process depending on the pipette pulling‐up speed, *v*. At the critical pulling‐up speed, *v*_c_, the nanowire growth mode changes from confined to non‐confined growth; when *v* ≤ *v*_c_ (confined growth mode), the nanowire diameter remains constant with *v*. In contrast, when *v* > *v*_c_ (non‐confined growth mode), the diameter decreases gradually with *v* due to the gradual stretching of the quasi‐liquid nanobridge. b) The diameter of DNA nanowires grown at *v* = 1, 2, 3, 5, 10, 15, and 20 µm s^−1^. The diameters over three nanowires at each condition were measured. The growth mode changes from confined (gray) to non‐confined mode (white) at *v*_c_. The field emission scanning electron microscopy (FE‐SEM) images display the five representative DNA nanowires depending on *v* (Scale bar, 1 µm). The diameters of the DNA nanowires grown under three tip diameters (*d*_t_ = 0.45, 0.82, and 1.36 µm) were measured depending on *v* (inset). The critical speed *v*_c_ was inversely proportional to *d*_t_ as fitted by the red line ($v_{c}\;=\;\frac{\alpha }{d_{t}}$). c) *vd*_t_ versus *d*/*d*_t_. The experimental coefficient, *α* ( = *v*_c_ *d*_t_), is marked by the vertical boundary line from confined growth (gray) to non‐confined growth (white). d) Dependence of *α* on the concentration and the length of DNA. *α* value is directly proportional to the concentration of DNA and inversely proportional to the square root of the molecular weight (∝length) of DNA. e) All the normalized critical speeds *v*_c_/*α* over various *C*, *M*, and *d*_t_ were well fitted to a single simulated line (black) equal to 1/*d*_t_.

Regarding the uniformity of the nanowire diameter, the pulling‐up speed of the nanopipette (*v*) determines two different modes of nanowire growth: Confined and non‐confined growth (**Figure** **2**a). A sufficiently low *v* prevents an abrupt shrinkage of the quasi‐liquid interface between the tip of the nanopipette and the top of the nanowire, thereby causing the nanowire diameter (*d*) to be confined by the tip diameter of the nanopipette (*d*_t_) (as confined growth). In contrast, a sufficiently high *v* stretches the nanobridge interface to form a conical meniscus, thereby gradually decreasing the nanowire diameter (as non‐confined growth). This suggests the existence of a critical pulling‐up speed (*v*_c_) at which the growth mode changes from confined to non‐confined growth. While the nanowire diameter remains constant at *d* = *d*_t_ in the confined growth mode (*v* ≤ *v*_c_), it can also be decreased with *v* under the non‐confined growth mode (*v* > *v*_c_).

We derived the critical pulling‐up speed of the pipette (*v*_c_) for the transition point from confined to non‐confined growth. In the confined growth mode, rapid solvent evaporation induces a continuous solution flow from the pipette tip. For a dilute solution, the solution flow rate is practically equal to the solvent evaporation rate at the quasi‐liquid nanobridge. Then,

(1) $$\frac{\pi }{4}d_{t}^{2}u\;=\;\pi d_{t}xE$$

where *u*, *x*, and *E* is the solution flow velocity, bridge length, and solvent evaporation flux, respectively. Here, the volume conservation of the solute in the bridge provides the following boundary condition:^[^ ^31^ ^]^

(2) $$u\approx \dot{g}\frac{\varphi -\varphi_{0}}{\varphi_{0}}$$

where $\dot{g}$ is the growth rate of the nanowire, and *φ* and *φ*_0_ are the volume fractions of the solute in the nanowire and pipette solutions, respectively. By combining Equations (1) and (2), we obtain:

(3) $$\dot{g}\;=\;\frac{4xE}{d_{t}}\frac{\varphi_{0}}{\varphi -\varphi_{0}}\;=\;\frac{\alpha }{d_{t}}$$

where the experimental coefficient (*α*),

(4) $$\alpha \;\left(\mathrm{experimental}\;\mathrm{coefficient}\right)=\frac{4xE\varphi_{0}}{\varphi -\varphi_{0}}$$

Notably, if the pulling‐up speed of pipette, *v*, is faster than the growth rate, $\dot{g}$, the liquid nanobridge cannot preserve its diameter and becomes stretched, thus, crossing over to the non‐confined growth mode. This indicates that the $\dot{g}$ obtained from Equation (3) is the maximum speed for confined growth, that is, *v*_c_ = $\dot{g}$. Therefore,

(5) $$v_{c}\;=\;\dot{g}\;=\;\frac{\alpha }{d_{t}}$$

Notably, *v*_c_ correlates with the concentration (*C*) and molecular weight (*M*) of the biopolymer solute in the solution. As *φ* >> *φ*_0_ in dilute solutions, from Equations (3) and (5), respectively, we obtain:

(6) $$v_{c}\;=\;\dot{g}\;\propto \;\varphi_{0}\;\propto \;C$$

Because the nanobridge length (*x*) is roughly correlated with the viscosity (*η*) of the solution,^[^ ^31^ ^]^

(7) $$x\;\propto \;\frac{1}{\sqrt{\eta }}$$

and the viscosity is correlated with the molecular weight,

(8) $$\eta \;\propto \;M^{\;n}\;$$

(*n*: Mark–Houwink parameter).^[^ ^32^ ^]^ Thus, from Equations (3), (5), (7), and (8), respectively, we obtain:

(9) $$v_{c}\;=\;\dot{g}\;\propto x\;\propto \frac{1}{\sqrt{\eta }}\;\propto \;\frac{1}{\sqrt{M^{\;n}}}$$

where *n* depends on the polymer‐solvent system. Further, from Equations (3)–(9), we obtain:

(10) $$v_{c}\;=\;\frac{\alpha }{d_{t}}\;=\;\frac{\kappa C}{\sqrt{M^{\;n}}}\propto \frac{1}{d_{t}}$$

where *κ* is a coefficient determined by the type of biopolymer and environmental factors.

As the pulling‐up speed increased, the growth mode crossed over from confined to non‐confined mode (Figure 2b and Figure S5, Supporting Information). When the DNA nanowire growth for *v* ≤ *v*_c_ was investigated with a given nanopipette (*d*_t_ = 0.82 µm), the nanowire diameter remained constant with *v* as *d* = *d*_t_, thus, indicating the confined growth (Figure 2b, gray). However, for *v* > *v*_c_, the nanowire diameter decreased gradually with *v* (*d* < *d*_t_), thus, indicating non‐confined growth (Figure 2b, white). The red line in the figure represents the simulated critical speed (*v*_c_), which is inversely proportional to *d*_t_ (based on Equation (5)). Although the length of the liquid nanobridge (*x*) in *α* ( = $\frac{4xE\varphi_{0}}{\varphi -\varphi_{0}}$ in Equation (4)) may be affected by the tip diameter of the nanopipette (*d*_t_), the dependency between *α* and *d*_t_ was almost negligible in the implemented range under 2 µm. Practically, upon measuring the diameter of DNA nanowires grown with different tip diameters (from *d*_t_ = 0.45–1.36 µm), the transition of the growth mode was observed at the red line (see the inset in Figure 2b), which indicates that *α* is independent of *d*_t_.

The estimation of *α* depending on experimental conditions, such as concentration, molecular weight, and type of biopolymer, facilitates precise control over the nanowire growth behavior. For example, we measured *α* for DNA nanowires grown at various concentrations (1, 2, and 4 mg mL^−1^) and base lengths (30, 60, and 120 nt for single‐stranded DNA [ssDNA]), where the normalized diameter (*d*/*d*_t_) was plotted depending on *vd*_t_ (Figure 2c). Here, *α* [ = *v*_c_ *d*_t_ by Equation (5)] is marked by the vertical boundary line from confined growth (gray) to non‐confined growth (white). Interestingly, the experimentally measured *α* values (Figure 2d, open circles) were proportional to *C* and 1/*M* ^*n*/2^, respectively, and matched with those calculated using Equation (10) (Figure 2d, dashed lines).

The Mark–Houwink parameter, *n*, was fitted to 1, which is consistent with a previous study on linear DNA,^[^ ^33^ ^]^ thus, indicating the high reliability of *α* determination.

From *v*_c_ identification and *α* value, we can easily predict nanowire growth modes, using Equation (10). When the DNA nanowires were grown at various ranges of *v* (1–20 µm s^−1^), *C* (1, 2, 4 mg mL^−1^), *M* (30, 60, and 120 nt for ssDNA), and *d*_t_ (450 nm–2 µm), the nanowire growth mode changed at the calculated *v*_c_, and all normalized critical speeds (*v*_c_/*α*) over various *C*, *M*, and *d*_t_ fitted well to a single simulated line equal to 1/*d*_t_ (Figure 2e). To confirm the universality of Equation (10), we elucidated the *α* values for different types of biopolymers (Figure S6a, Supporting Information). We performed 3D writing with different biopolymers, such as bovine serum albumin (BSA) protein and dextran. Irrespective of the type of biopolymer used, the transition of the growth mode appeared at the critical speed *v*_c_, which could be normalized as *v*_c_/*α* (black line, Figure S6b, Supporting Information). Our observations suggest that regardless of the concentration (*C*), molecular weight (*M*), and type of biopolymer (*κ*) applied, the identification of *v*_c_ enables rational design of the growth of biopolymer nanowires with uniform diameters.

### 2.3 Multiplexed Biopolymer Nanowire Arrays Fabricated by 3D Nano‐Patterning

**Figure 3:**
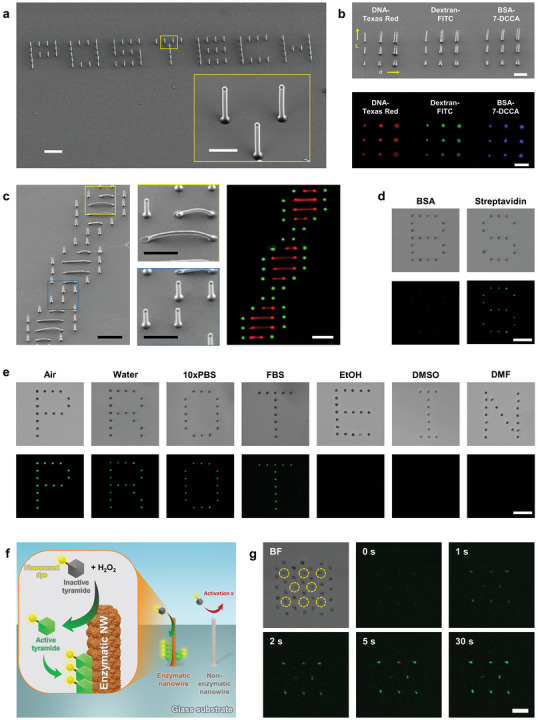
On‐demand fabrication of 3D biopolymer architectures. a) Field emission scanning electron microscopy (FE‐SEM) image of a nanowire pattern consisting of homogeneous freestanding nanowires. Scale bar, 20 µm. (inset: Scale bar, 5 µm). b) FE‐SEM (top) and confocal (bottom) images of 3D nanowire pattern with individually controlled length (6–18 µm), diameter (0.5–2 µm), and biopolymers [DNA‐Texas Red (red), Dextran‐FITC (green), and BSA‐7‐DCCA (blue)], grown on a glass substrate. Scale bar, 10 µm. c) A complex 3D pattern composed of shape‐controlled DNA nanowires demonstrates the controllability of 3D shapes, such as arch (yellow inset) and pillar (blue inset) (left). The nanowires consisting of DNA conjugated with Texas Red (red) and FAM (green) are site‐specifically positioned, as shown in the confocal image (right). Scale bar, 20 µm. (inset: Scale bar, 10 µm). d) Streptavidin (left) and bovine serum albumin (BSA) (right) nanowire patterns were immersed in FITC‐biotin solution for 10 min. Scale bar, 10 µm. The bright (top) and dark (bottom) field images demonstrate the shape retention of the nanowire patterns in 1 × phosphate‐buffered saline (PBS) solution and also the function retention of enclosed biopolymers. e) The bright (top) and dark (bottom) field images of the streptavidin nanowires in different solvent environments of air, water, 10 × PBS, FBS, ethanol, DMSO, and DMF, representing high mechanical and functional stability. Scale bar, 10 µm. f) Schematic illustration of enzymatic nanowire that induces tyramide signal amplification. On the surface of the enzymatic nanowire, the tyramide reagent is activated by enzyme‐induced peroxidation and is immediately deposited on the nanowire surface. g) Real‐time monitoring of pin‐point enzymatic response in the protein‐based nanowire pattern. The bright field image shows the top view of the nanowires where the enzymatic nanowires (yellow circles) are located at the center of the hexagonally arranged non‐enzymatic nanowires. Dark field images show the catalytic reactivity of the arranged enzymatic nanowires demonstrated by the deposition of fluorescent tyramide agents for 30 s. Scale bar, 10 µm.

Based on 3D writing in confined growth mode, we successfully fabricated diverse 3D nanowire patterns with various dimensions, geometries, and materials. Homogeneous freestanding nanowires were reproducibly and site‐specifically grown with a uniform diameter and length (**Figure** **3**a). On a Si substrate, seven different character patterns were constructed using double‐stranded DNA (120 bp) nanowires of *d* = 850 nm and *l* = 10 µm, with each nanowire located 10 µm apart (see inset). As different biopolymers could be readily used to form uniform nanowires, three distinct dye‐conjugated biopolymers [DNA‐Texas Red (red), Dextran‐FITC (green), and BSA‐7‐DCCA (blue)] were demonstrated to form nanowires with individually controlled length (6–18 µm) and diameter (0.5–2 µm) by steering the fine‐tuned nanopipettes (Figure 3b). The precise control of nanopipette movement guided the direction of nanowire growth, thereby allowing wiring construction through the interconnection of two different points. Accordingly, we manufactured a complex 3D pattern that combines curved arches and freestanding pillars at the designated positions on a glass substrate (Figure 3c). The curved arches with specific widths of 10–20 µm were placed at regular intervals of 10 µm using DNA‐Texas red (red), and the pillars consisting of DNA‐FAM (green), with a uniform length of 10 µm, were printed along the edge of the DNA arches.

Even after 3D nano‐patterning, the freestanding biopolymer nanowires retained their inherent molecular functions, such as molecular recognition and bio‐catalysis, whereas their mechanical stability and structural integrity could be further enhanced via simple post‐modification (Movies S3 and S4, Supporting Information); when the 3D nano‐patterns were simply immersed in the organic solvent that contained crosslinkers, the surficial biopolymers of the nanowires could be exclusively interconnected to retain their structural shapes even in water. When two different protein nanowires, comprising pure streptavidin (Figure 3d, left) and BSA (right), respectively, were developed to maintain the freestanding structures in an aqueous solution, their unique target binding and antifouling abilities were well conserved. In the presence of 100 nM FITC‐biotin, the positions of streptavidin nanowires were fully overlapped with those of green fluorescence signals due to the specific streptavidin‐biotin non‐covalent interaction,^[^ ^34^ ^]^ whereas nothing was bound to the nanowires of BSA, which is one of the most effective antifouling proteins in the blood.^[^ ^35^ ^]^ Importantly, the protein nanowires could retain their original shapes and functionalities in various solvent environments. When immersed in deionized water, high‐salt buffer (10 × phosphate‐buffered saline), physiological solution (fetal bovine serum (FBS)), and frequently used organic solvents (ethanol, dimethyl sulfoxide (DMSO), and dimethylformamide (DMF)), the streptavidin nanowires underwent no structural deformation (Figure 3e, top), thus, enabling the localization of FITC‐biotin (bottom). Notably, the fluorescence intensities varied depending on the solvent environment, presumably proportional to the quantum yield of FITC in each solvent (Figure S7, Supporting Information).

At the submicron scale, elaborate patterning of proteins, such as receptors, antibodies, and enzymes, would be of great interest due to the requirement of site‐specific target recognition or metabolic regulation in controlling biological systems.^[^ ^36^ ^]^ As site‐specific localized chemical cues have application potential in biological studies, such as cell signaling and organelle mimicking,^[^ ^37^ ^]^ we prepared an enzymatic nanowire pattern based on horseradish peroxidase (HRP) (Figure 3f). To confirm the HRP‐accelerated peroxidation at local points, we used tyramide reagents; unlike easily diffusible reagents, such as 2,2′‐azino‐bis(3‐ethylbenzothiazoline‐6‐sulfonic acid)^[^ ^38^ ^]^ and luciferin,^[^ ^39^ ^]^ they can be immediately deposited at the location of peroxidation, thereby allowing real‐time reporting of catalytically‐active sites with high precision.^[^ ^40^ ^]^ Enzymatic and non‐enzymatic nanowires were densely printed with HRP and streptavidin on a glass substrate, and Alexa Fluor‐488 tyramide and H_2_O_2_ were introduced to initiate pin‐point peroxidation reactions (Figure 3g). Green fluorescence was observed only on the surface of the enzymatic nanowires (yellow circles), and the fluorescence signals increased logarithmically with time and saturated at 30 s (Figure S8, Supporting Information), probably because of the complete occupation of the tyramide deposition sites. However, the fluorescence of the adjacent non‐enzymatic nanowires was negligible, indicating the availability of enzymatically active 3D‐shaped satellites in one batch reaction. Ultimately, such protein‐based nanopatterns enabled spatiotemporal control of chemical and physical stimuli with submicron resolution, thus, potentially serving as artificial cellular organelles such as peroxisomes underneath live cells and tissues.^[^ ^41^ ^]^

## 3 Conclusion

By exploiting the nanoscale confinement of the biopolymer solution in the quasi‐liquid nanobridge, we achieved direct 3D writing and nano‐patterning of biopolymers. Additionally, through precise control over nanowire growth, we reproducibly fabricated freestanding nanowires and arch‐type wiring. The diameter of the nanowires was adjusted in the range 80 nm–10 µm, which could be further extended by fine‐tuning the glass pipettes.^[^ ^42^ ^]^ Based on the minimum distance of 2 µm between the nanowires (Figure S9a, Supporting Information), their length can be even shortened to be 1 µm (Figure S9b, Supporting Information) or elongated to more than a few millimeters (Figure S10, Supporting Information); although the gradual increments of 250 nm in distance and length were achievable by the spatial resolution of 3‐axis motor stage, the maximum distance and length were limited by its z‐axis travel range. As the composition of the nanowire is simply determined by the choice of biopolymer solution, different biopolymers of a single or several mixed components could be readily printed at the desired position on a chosen substrate and even on the top of pre‐printed wires (Figure S11, Supporting Information). Currently, the 3D writing only produces one nanowire at a time and writing speed under the confined growth mode is quite slow (<10 µm s^−1^); however, parallel printing of biopolymer nanowires might improve throughput with the development of well‐aligned multi‐channel nanopipettes.^[^ ^43^ ^]^ While the process of 3D writing is exceptionally simple in ambient air, the molecular orientation of biopolymers has not been controlled, causing nanowires to possess amorphous characteristics. However, the recent advancement of self‐assembly techniques using external stimuli (such as ultrasound^[^ ^44^, ^45^ ^]^ and pulsed electric field^[^ ^46^ ^]^) may be synergistically combined with our 3D writing of confined biopolymers to facilitate full control over the crystallinity of the printed architectures by varying the mechanical and biochemical properties.

As proteins are involved in most cellular functions and phenotypes in all living species, their spatially controlled 3D immobilization is crucial for sensing, signaling, and transporting small biological entities in microscopic environments. Existing techniques, such as laser‐assisted printing^[^ ^47^ ^]^ or micro‐molding,^[^ ^48^ ^]^ frequently require full crosslinking of internal structures for structural miniaturization and shaping of desired architectures, but high‐level modification inevitably reduces the functional integrity of embedded biopolymers.^[^ ^25^, ^49^ ^]^ In contrast, our method enables free‐form structuring of different biopolymers and exclusive modification of the surface of the pre‐built nanostructures at mild conditions, thereby reducing the risk of functional loss, but enhancing the mechanical stability and environmental tolerance. Fully intact biopolymers can be compartmentalized by crosslinked surface networks that serve as size‐exclusive membranes for the materials approaching the inside of the nanowire, thereby realizing the potential for mimicking microvascular channels with capillary exchange function.^[^ ^50^ ^]^ Moreover, depending on the type of crosslinker, photodegradable or biodegradable biopolymer architectures can be created, which would be essential in the production of nanoneedle patches for intracellular drug delivery. Given the technical availability of high‐throughput 3D nano‐patterning and hierarchical nanostructuring (e.g., core–shell formation), our type‐independent biopolymer printing technique can help fabricate different 3D biopolymer ensembles with application potential in several technologies, such as organ‐on‐chips.

## 4 Experimental Section

### Materials

BSA, Dextran, fluorescein isocyanate dextran (Dextran‐FITC), 7‐(Diethylamino) coubarin‐3‐carboxylic acid *N*‐succinimidyl ester (7‐DCCA NHS), *N*‐ethyl‐*N*’‐(3‐dimethylaminopropyl) carbodiimide hydrochloride (EDC), hexamethylene diamine, disuccinimidyl suberate, agar powder, biotin‐4‐fluorescein (FITC‐biotin), streptavidin‐horseradish peroxidase conjugate (Streptavidin‐HRP), pH 8.5 Tris‐HCl buffer, hydrogen peroxide, 10 × phosphate buffered saline (PBS), ethanol, DMSO, *N*,*N*‐dimethylformamide, and 1‐methyl‐2‐pyrrolidinone (NMP) were purchased from Sigma‐Aldrich (St. Louis, MO). FBS was purchased form Gibco (Waltham, MA). Streptavidin was purchased from Promega (Madison, WI). Alexa Fluor‐488 Tyramide was purchased from Thermo Fisher Scientific (Waltham, MA). Deionized water (18MΩ) was used in all experiments. Lambda DNA and DNA oligonucleotides were synthesized by Bioneer (Daejeon, Korea) (Table S1, Supporting Information).

### *3D* Writing

3D writing of biopolymers was performed using a customized setup consisting of a print head with a solution‐filled nanopipette and a building platform with glass or silicon substrate. For a nanopipette preparation, borosilicate glass capillaries (BF‐100‐50‐10, Sutter Instrument) were tapered by flame‐brushing process where the diameter of pipette tip was precisely controlled by programmed heating and pulling conditions (P‐97 micropipette puller, Sutter Instrument). To fabricate a nanowire array, the position of the nanopipette and the substrate were spatially controlled by 3‐axis motor stage with an accuracy of 250 nm (XA07A and ZA07A, Kohzu Precision). The fabricated nanopipette was filled with a biopolymer solution of which concentration was ranging from 0.5 to 4 mg mL^−1^ in deionized water, and then it was subsequently pulled down closely to the substrate, thereby forming a quasi‐liquid nanobridge in‐between. Thereafter, the nanopipette was pulled with programmed speed (>0.5 µm s^−1^) and direction to produce a biopolymeric nanowire of desired shape, and the pipette was rapidly separated to liberate the nanowire on the substrate (Movie S1, Supporting Information). The nanowire fabrication process was monitored in real‐time with the customized optical imaging system consisting of CCD cameras (INFINITY 1–2C, Lumenera Camera), objective lens (100x Plan Apo Infinity Corrected Objective, Mitutoyo), and 590 nm LED light source (Precision LED spotlight, Mightex).

### Characterization of Biopolymer Nanowires

The crystallinity of biopolymer molecules within the fabricated nanowire was inspected by using a TEM (JEM‐2100F, JEOL) operated at 80 kV. The spatial elemental distribution in nanowires was investigated by EDS‐mapping operated at 80 kV of a fifth‐order aberration(C_s_)‐corrected STEM (JEM‐ARM200F, JEOL). For the TEM sample preparation, the biopolymer nanowires (*d* = 89 ± 5 nm (mean ± s.d.)) were directly fabricated in the lateral direction on a lacey carbon film supported copper TEM grid (Figure S1 and Movie S2, Supporting Information). For the investigation of nanowire growth behavior, the diameters of nanowires grown at different printing speeds were measured with field emission scanning electron microscopy (FE‐SEM, S‐3400N, Hitachi). The appearances of nanowire patterns were characterized by using a high‐resolution FE‐SEM (JSM‐7800F Prime, JEOL). The fluorescent imaging of the fabricated nanopatterns was performed using a confocal microscope (STELLARIS 5, Leica).

### Real‐Time Polymerase Chain Reaction (RT‐PCR) Analysis

Using nanopipettes filled with the ssDNA‐120 solution (25 µM in water), nanowires were grown on tapered optical fiber tips with the constant pulling speed of 1 µm s^−1^. The tapered optical fibers were prepared by using a laser‐based puller (P‐2000, Sutter instrument). To identify the proportionality between the volume of nanowire and the number of DNA molecules in the nanowire, four different nanowires at varying lengths (5, 10, 20, and 40 µm) (Figure S3a, Supporting Information) were prepared, and then, they were dissolved in water (2 µL). Thereafter, the number of DNA molecules were quantified by RT‐PCR (LightCycler 480, Roche). Each sample for RT‐PCR reaction (20 µL) contained 0.2 µL of F‐primer (50 µM), 0.2 µL of R‐primer (50 µM), 2 µL of DNA‐dissolved water, 10 µL of LightCycler 480 SYBR green I master mix, and 7.6 of nuclease‐free water (T&I). Prepared samples were subjected to PCR cycles (20 s denaturation at 94 °C, 20 s annealing at 57 °C, and 20 s extension at 72 °C).

### Gel Electrophoresis

To investigate structural integrity of biopolymer molecules after 3D writing process, the lengths of DNAs in microwires, consisting of single types of DNA molecules (ssDNA‐30, ssDNA‐60, ssDNA‐120, dsDNA forms of ssDNA‐120, and lambda DNA, respectively) were analyzed by gel electrophoresis. For this assay, dsDNA forms of ssDNA‐120 were prepared by obtaining PCR‐amplified products of ssDNA‐120 (PCR condition: 30 s denaturation at 95 °C, 1 min annealing at 57 °C, and 1 min extension at 72 °C), and then, PCR products were purified by using QIAquick PCR purification kit (QIAGEN). The other oligonucleotides were purchased from Bioneer as mentioned above. Each microwire was fabricated with the constant diameter (5 µm) and length (900 µm) on a tapered optical fiber tip (Figure S10, Supporting Information), and dissolved in the 3 µL solution with 2 µL of water and 1 µL of 6x loading buffer (T&I). All single‐stranded oligonucleotides were resolved on an 8 m urea‐10% PAGE‐1X TBE gel, and the others (dsDNA forms of ssDNA‐120 and lambda DNA) were resolved on a 10% PAGE‐1X TBE gel. Gel images were acquired by Azure c600 (Azure Biosystems).

### Post‐Crosslinking of Protein Nanowires

To perform crosslinking reaction, the external surface of protein was cationized by carbodiimide‐activated conjugation of hexamethylene diamine. For this, 9 mg hexamethylene diamine was dissolved in water and adjusted to pH 6.5 using 1 m HCl. Subsequently, a solution of BSA (1 mg) or streptavidin (1 mg) was mixed with the hexamethylene diamine solution, and then, 3 mg EDC was added immediately. After 6 h stirring at room temperature, the solution was subjected to molecular weight filtration by using molecular weight cutoff filter (30 kDa MWCO) to remove excess unreacted reagents. The obtained product was freeze‐dried and dissolved in 50 mm Tris‐HCl buffer (pH 8.5) before nanowire fabrication. After manufacturing nanowire patterns with cationized protein solutions, the patterns were immersed in the solution of disuccinimidyl suberate (100 mm in NMP) for 5 min to produce supramolecular protein network on the surface of nanowire. The obtained nanowire patterns were cleaned with NMP and water. To deactivate remaining *N*‐hydroxysuccinimide (NHS) ester groups on the nanowire surface, the nanowire patterns were immersed in ethanol amine solution (100 mm, pH 9) and rinsed with 10 × PBS and water. Mechanical enhancement of the crosslinked protein nanowire was evaluated by its insertion into 1 wt% agarose gel. Without crosslinking, the shape of protein nanowire was deformed near the moist surface of the agarose gel, and dissolved after insertion into the gel (Movie S3, Supporting Information), but the crosslinked nanowires exhibited improved mechanical strength enough to retain its shape after their insertions into the gel (Movie S4, Supporting Information).

## Conflict of Interest

The authors declare no conflict of interest.

## Supporting information

Supporting Information

Supplemental Movie 1

Supplemental Movie 2

Supplemental Movie 3

Supplemental Movie 4

## Data Availability

The data that support the findings of this study are available from the corresponding author upon reasonable request.
