# Supplementary material for: Type‐Independent 3D Writing and Nano‐Patterning of Confined Biopolymers
Source: Adv Sci (Weinh). 2023 Feb 24;10(13):2207403. doi: 10.1002/advs.202207403 (PMC10161081; doi:10.1002/advs.202207403)
Supplement: Supplementary file 1 — Supporting Information [file ADVS-10-2207403-s001.pdf]

Supporting Information

Type-Independent 3D Writing and Nano-Patterning of Confined Biopolymers

Un Yang, Byunghwa Kang, Moon-Jung Yong, Dong-Hwan Yang, Si-Young Choi, Jung Ho Je, and Seung Soo Oh***

U. Yang, B. Kang, M. J. Yong, D. H. Yang, S. Y. Choi, J. H. Je, and S. S. Oh
Department of Materials Science and Engineering, Pohang University of Science and Technology (POSTECH), 77 Cheongam-Ro, Nam-Gu, Pohang, Gyeongbuk 37673, South Korea.
E-mail: jungho.je@nanoblesse.com (J. H. Je) and seungsoo@postech.ac.kr (S.S. Oh)

S. S. Oh
Institute for Convergence Research and Education in Advanced Technology (I-CREATE), Yonsei University, 85 Songdogwahak-ro, Yeonsu-gu, Incheon, 21983, South Korea

J. H. Je
Nanoblesse, 85-11 (4th fl.) Namwon-Ro, Pohang, 37883, South Korea

Keywords: biopolymers, 3D writing, nanoscale confinement, solvent-exclusive evaporation, sub-micron resolution

Supporting Figures and Tables

Table S1. Oligonucleotides used in this work

Name	Sequence (5' to 3')	Length (nts)	Molecular weight (Da)
ssDNA-30	CTC CAA CAT CAA GGA AGA TGG CAT TTC TAG	30	9278.03
ssDNA-60	AAT TCT GGG GGA GCC TTT TGT GGG TAG GGC GGG TTG GTT TTG	60	18831.16

	CCC CGG AGG AGG AAT TTC		
ssDNA-120	CAA CTT CCT CAA GGA ACA ACA TTG CCA AAA GGC TTC TAC GCA GAG GGG AGC AGA GGC GGC AGT CAA GCC TCT TCT CGC TCT TCA TCA CGT AGT CGC GGT AAT TCA AGA AAT TCA ACT CCT	120	36966.93
F-primer	CAA CTT CCT CAA GGA ACA AC	20	6117.97
R-primer	AGG AGT TGA ATT TCT TGA AT	20	6265.10
DNA-Texas Red	[Texas Red] CTC CAA CAT CAA GGA AGA TGG CAT TTC TAG	30	10094.97
DNA-FAM	[FAM] TTT TTT CAG TCA GTC TAC GC	20	6971.91

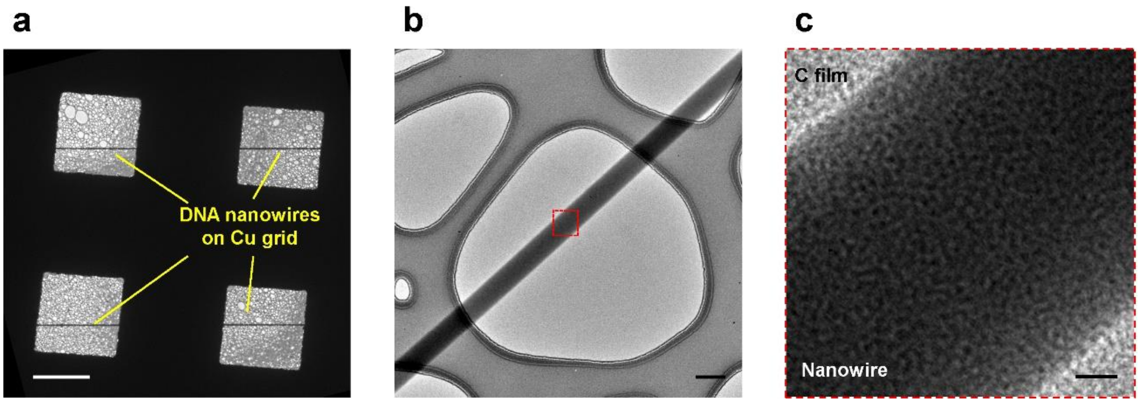

Figure S1. TEM images of lambda DNA nanowires. **a,** The DNA nanowires laterally fabricated on a copper grid. Scale bar, 10 µm. **b,** A bright-field TEM image of DNA nanowire. Scale bar, 100 nm. **c,** High resolution TEM image of red box in **b**. The nanowire exhibits amorphous characteristic with the diameter of 89 ± 5 nm (mean ± s.d.). Scale bar, 10 nm.

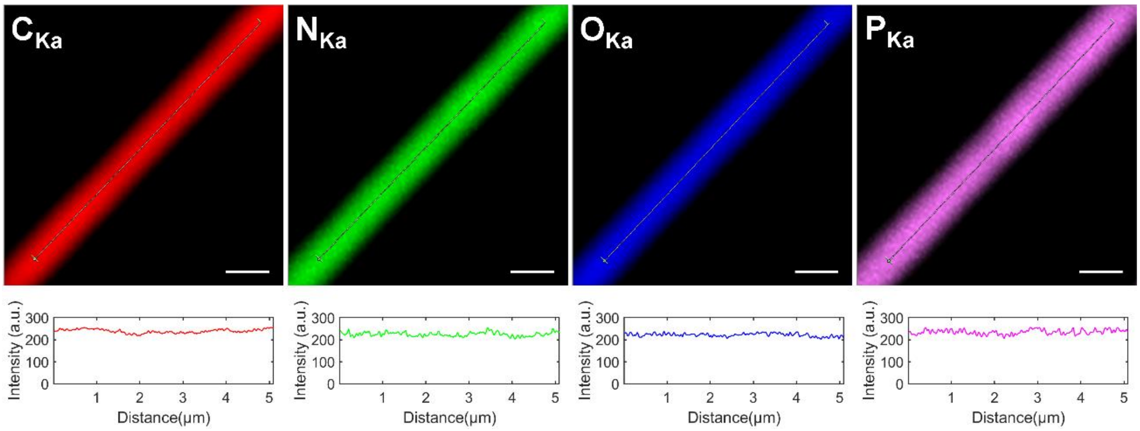

Figure S2. STEM-EDS images of a lambda DNA nanowire and their intensity profile along the line segment in the images. The DNA nanowire grown by our direct 3D nanowriting has the uniform distribution of DNA-composing elements: carbon (red), nitrogen (green), oxygen (blue), and phosporus (margenta). Scale bar, 100 nm.

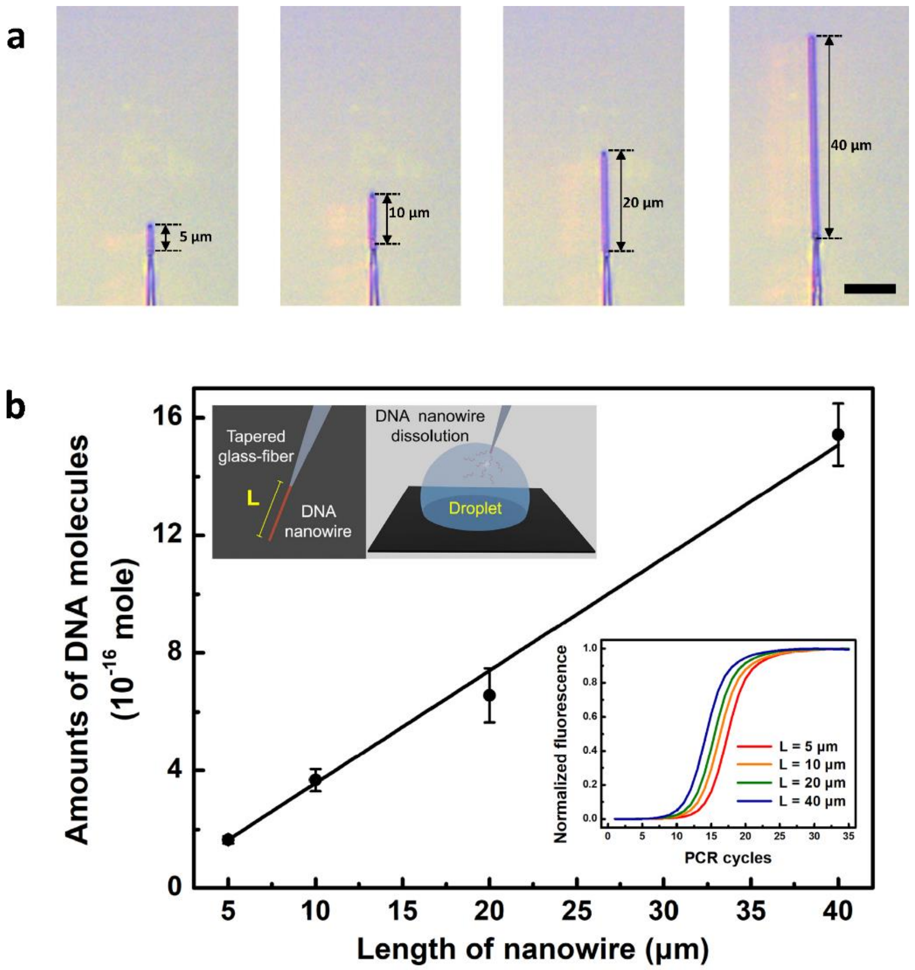

Figure S3. Quantification of the number of DNA molecules in nanowires with four different lengths (5, 10, 15, and 20 μm). **a**, DNA nanowires with different lengths, fabricated on tapered optical fiber tips. The length of the nanowire can be precisely adjusted by controlling the pulling-up distance of the nanopipette during our 3D writing process. Scale bar, 10 μm. **b**, Quantitative analysis of DNA molecules in nanowires. The simple variation of the nanowire length allows accurate quantification of DNA even at sub-femtomole scale, evidenced by RT-PCR reaction after dissolving nanowires in distilled water. Left inset: schematic illustration showing a DNA nanowire grown on a tapered optical fiber tip and its dissolution into water droplet. Right inset: PCR amplification curves obtained from the DNA nanowires dissolved in water, indicating the increase in the amount of DNA molecules depending on the nanowire length.

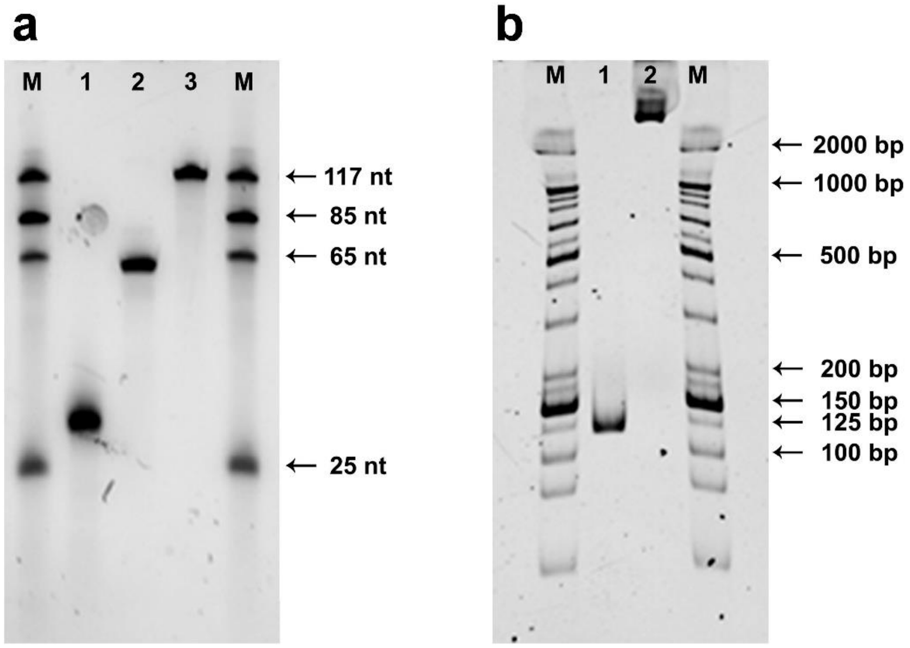

Figure S4. Gel electrophoresis analysis of DNA molecules in DNA nanowires. The single band in each lane demonstrates no chemical degradation of DNAs in the nanowires due to the mild processing environment of room temperature and ambient air. **a**, Lane M, DNA ladder; lane 1, ssDNA-30; lane 2, ssDNA-60; lane 3, 120 ssDNA-120. **b**, Lane M, DNA ladder; lane 1, dsDNA forms of ssDNA-120; lane 2, lambda DNA. bp is the abbreviation of base-pairs.

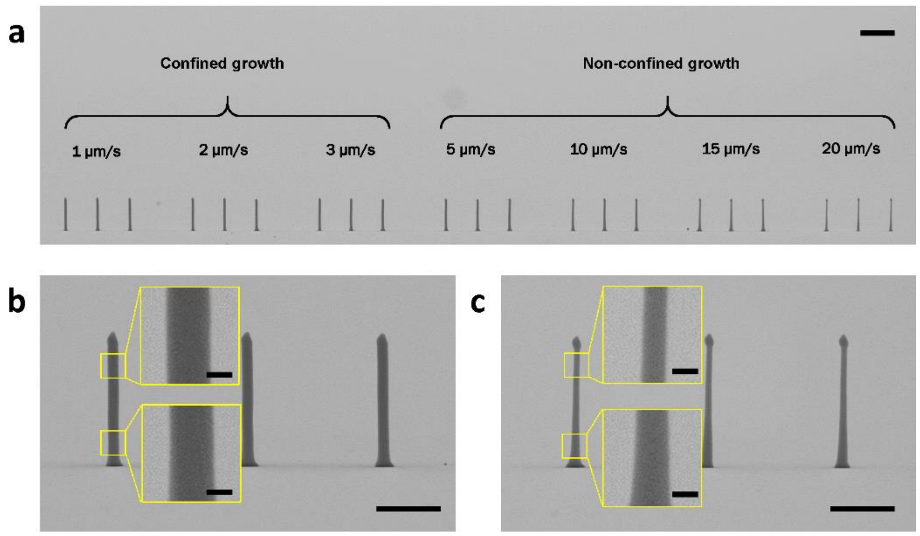

Figure S5. FE-SEM images of DNA nanowires. **a**, DNA nanowires grown at v = 1, 2, 3, 5, 10, 15, and 20 µm/s. Scale bar, 10 µm. At low v (= 1, 2, and 3 µm/s), the nanowire diameter remains constant with v (*confined* growth mode). At high v (= 5, 10, 15, and 20 µm/s), the nanowire diameter is gradually getting smaller with v (*non-confined* growth mode). **b**, DNA nanowires grown at v = 1 µm/s (*confined* growth). The diameters of the nanowires grown in confined mode are uniform. Scale bar, 5 µm. (inset: Scale bar, 500 nm). **c**, DNA nanowires grown at v = 20 µm/s (non-confined growth). The diameters of the nanowires grown in non-confined mode are not uniform up to certain heights, in particular near the nanowire foots, mostly due to the initial stretching of the nanobridges on the substrate. Scale bar, 5 µm. (inset: Scale bar, 500 nm).

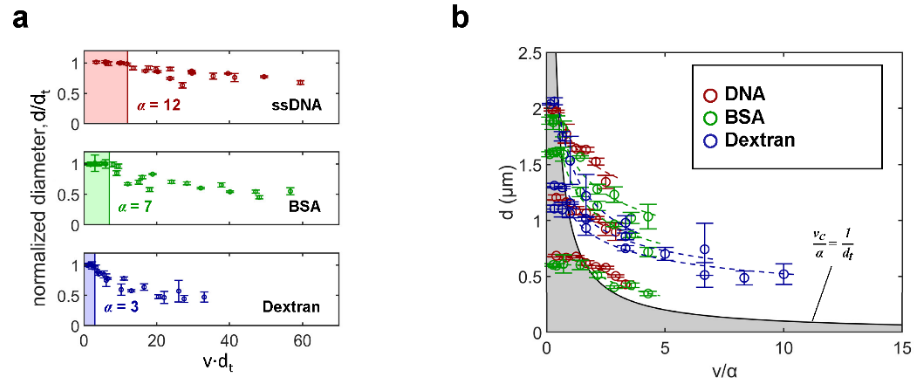

Figure S6. Growth dynamics of biopolymer nanowires with three different types of biopolymers (DNA, BSA, and Dextran). **a**, vd_t vs d/d_t. The experimental coefficient α corresponds to $v_c d_t$. **b**, All the normalized critical speeds v_c/α over 3 types of biopolymers are well fitted to a single simulated line (black) which is equal to $1/d_t$.

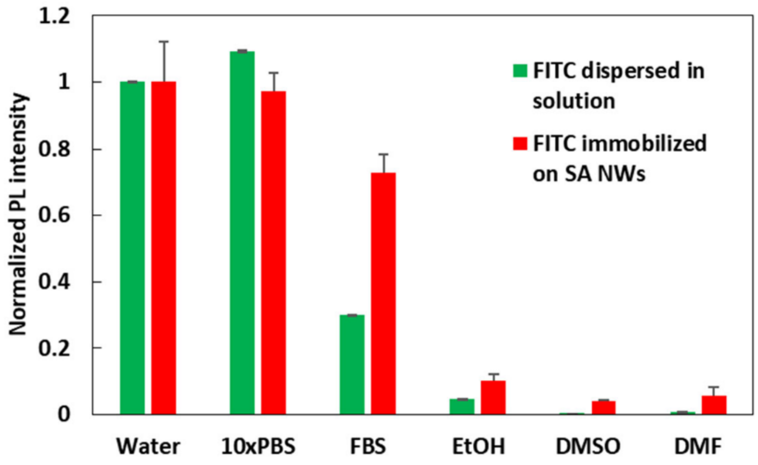

Figure S7. Solvent-dependent fluorescent intensity of FITC. The normalized photoluminescence (PL) intensities were measured for FITC dispersed in solution (green) and FITC immobilized on streptavidin nanowires (red), respectively, in different solvent (water, 10× PBS, FBS, ethanol, DMSO, and DMF). The PL intensity of the FITC-biotin solution was measured by UV-visible spectrometer (RF5301 PC, SHIMADZU) (excitation wavelength: 488 nm). To measure the PL intensity of the FITC-biotin immobilized on the nanowires, the streptavidin nanowire patterns on glass substrates were immersed in each solvent, and fluorescence confocal images were obtained by using confocal microscope (STELLARIS 5, Leica) with the excitation laser wavelength of λ = 488 nm.

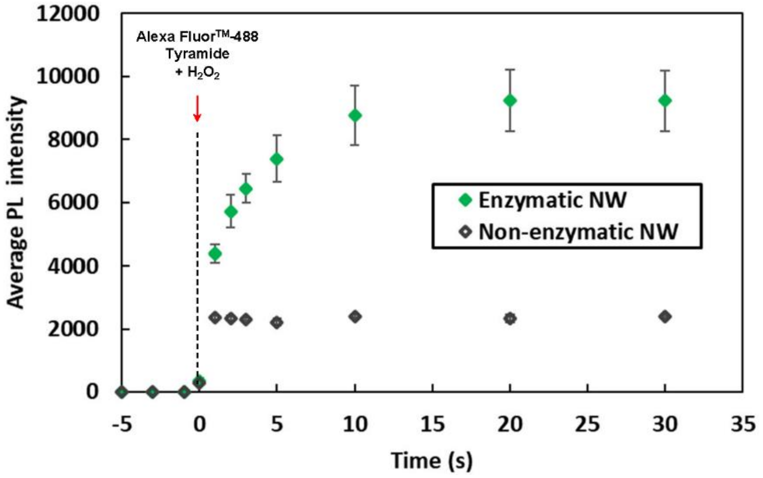

Figure S8. Real-time peroxidase activity monitoring of localized protein nanowires. After injection of Alexa Fluor^{TM}-488 Tyramide and H_2O_2 (t = 0 s), the PL intensity gradually

increased with time for 30 s on the surface of the enzymatic nanowires, but became constant on that of non-enzymatic nanowires.

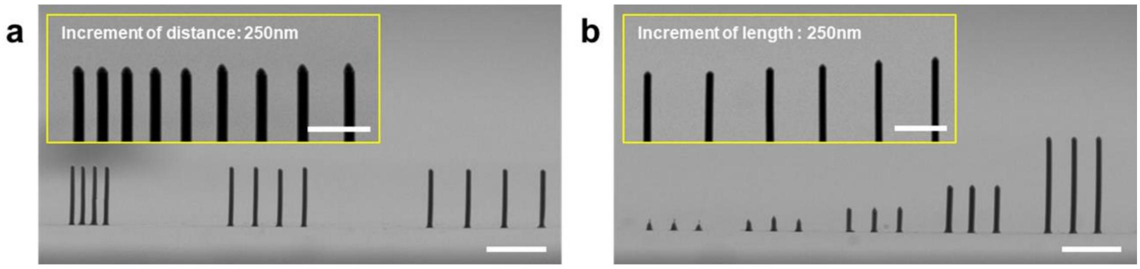

Figure S9. Evaluation of nanowire distance and length controllability. **a**, DNA nanowires fabricated with the controlled distance of 2, 4, and 6 μm, respectively. Given the current conical shape of nanopipette, the minimum distance between the DNA nanowires was 2 μm. Scale bar, 10 μm. (inset: DNA nanowires fabricated with the increment of 250 nm in distance. Scale bar, 5 μm). **b**, Length control of DNA nanowires (1, 2, 4, 8, and 16 μm). The length of nanowires was precisely determined by controlling the upward pulling distance of nanopipette using a z-axis motor stage. Scale bar, 10 μm. (inset: DNA nanowires fabricated with the increment of 250 nm in distance. Scale bar, 5 μm).

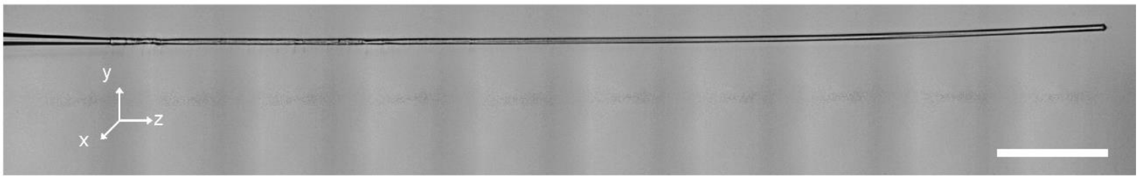

Figure S10. A DNA microwire fabricated on a tapered optical fiber tip. The length can be elongated to be more than a few millimeters, just limited by the travel range of the z-axis motor stage for pulling-up of the pipette. Scale bar, 100 μm.

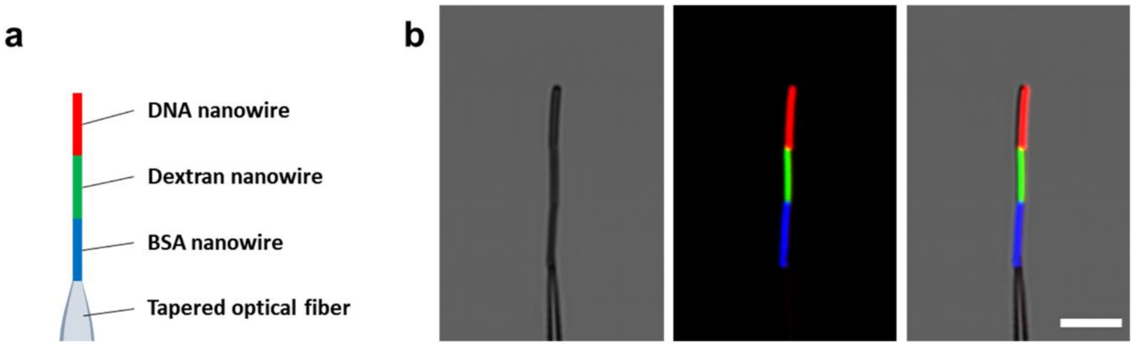

Figure S11. Vertical stacking of biopolymeric nanowires. **a**, Schematic illustration of the hybrid nanowire composed of three different biopolymers (DNA-Texas Red (red), Dextran-FITC (green), and BSA-7-DCCA (blue)). **b**, The bright field (*left*), dark field (*middle*), and their merged (*right*) image of the vertically stacked nanowire. By the accurate alignment of nanopipette, another nanowire can be grown on the top of the pre-fabricated nanowire. Scale bar, 10 μm.

Captions for Movies S1 to S4

Movie S1. 3D writing process for fabricating biopolymer nanowires.

Movie S2. Fabrication of a lambda DNA nanowire on a Cu TEM grid.

Movie S3. Streptavidin nanowire insertion into 1wt% agarose gel before post cross-linking.

Movie S4. Streptavidin nanowire insertion into 1wt% agarose gel after post cross-linking.
